# Effects of Zymotic Diseases upon the Teeth

**Published:** 1885-09

**Authors:** F. S. Whitslar

**Affiliations:** Youngstown, Ohio


### EFFECTS OF ZYMOTIC DISEASES UPON THE TEETH.

BY DR. F. S. WHITSLAR, YOUNGSTOWN, OHIO.

READ AT THE MEETING OF THE NORTHERN OHIO DENTAL SOCIETY, HELD IN YOUNGSTOWN, O., MAY 13, 1885.

In the consideration of this subject we are brought to the investigation, not only of the causes of decay of the teeth, but of the arrest of the developmental process. Causes of decay are two-fold, and may be classed as predisposing and exciting. You will bear in mind that these two divisions are clear and distinct. There are many things which predispose teeth to decay, but we know more of the exciting than of the predisposing causes. When the structure is imperfect, they are more likely to decay than where they are perfect. Then again, the teeth may be comparatively well organized, but owing to defective nutrition and enfeeblement of the vital forces, they are more readily susceptible to decay. With regard to exciting causes, there has been much discussion. Grouping the various opinions entertained by those who have experimented and written extensively upon the subject, we have:

1st, Those who regard it as a real disease, a vital phenomenon strictly comparable to morbid conditions of other more highly organized parts of the body.

2d, Those who regard it in the main as the effect of mere chemical action, but who also consider that some very constant appearances are only explicable on the hypothesis of vital action.

3d, Those who consider it entirely the effect of chemical action, in no degree modified by connection with a living organism. Fox, Bell, Hunter, Cuvier, Hertz, and Neuman maintained that dental caries was an inflammatory affection, a true disease of the dentine, and the name of "Odontitis" was given to this supposed disease. This theory has so much evidence against it, and so little in its favor, that it need not detain us longer.

As preliminary to what I shall say, I wish to call attention to the constituent elements of the teeth, and the bonds which hold them together. Of the sixteen elements composing the human body, about twelve are found in the structure of the teeth, and they may be classed into organic and inorganic matter. The bonds which hold together or maintain the structural integrity of the teeth are first, the vital, second the chemical, and third, the mechanical, the latter depending upon the first two. Remove the vital principle from a tooth, and the chemical force soon begins to be impaired, and disintegration from the first is comparatively an easy process. The vital force, when present, holds the peculiar chemical affinities that exist here in its grasp, and the latter are only at their best when thus governed.

What are zymotic diseases? Dunglison informs us that a zymotic disease is any epidemic, endemic, contagious, or sporadic affection, which is produced by some morbific principle acting on the organism similar to a ferment, as the major exanthemata. In the classification of Dr. Wm. Farr, zymotic diseases comprise diseases which are epidemic, communicable, inoculable, capable of propagation from existing force, or by the want or bad quality of food. This class includes four orders: Miasmatic, enthetic, dietic, and parasitic.

The first thing to which we ask your attention as a probable effect of zymotic diseases upon the teeth is the partial arrest of their development. The crowns of teeth bearing the marks of interrupted development, instead of presenting the smooth and glassy surface characteristic of finely developed enamel, are disfigurèd by the presence of an irregularly grooved and pitted surface, usually accompanied by a considerable diminution in size. The incisors are commonly very thin and compressed, while the cuspids and cusps of the molars are terminated by sharp points. The enamel and dentine tissues are not only deficient in quantity, but are defective in quality. The enamel will be porous, yellow, opaque, and fragile, while in dentine, that uniformity of size and arrangement of the dentinal tubes which they exhibit in well developed teeth is wanting. The defect of structure will be found limited to such portions of the several teeth as were undergoing development at the same time, and consequently under the same constitutional conditions. We sometimes find teeth which are marked by grooves and ridges regularly disposed. The grooves are the result of imperfect, and the ridges of perfect, development of the enamel and subjacent dentine. These transverse markings, resulting from alternations in the developmental process, find almost an exact parallel in the striæ produced by similar causes on the nails. This parallel points to the theory that the teeth are appendages of the skin, and belong to dermal or tegumentary tissues. You are doubtless familiar with the statement of

Tomes, that "The mucus membrane which lines the alimentary canal is continuous with, is indeed a part of, the external skin with which it blends at the lips, and that all teeth are alike developed from a part of the mucus membrane, and any connection which they may ultimately get with the bone is a secondary matter."

Salter says: "Teeth certainly are not extraneous organs, as suggested by Hunter, but have a distinct vitality. They undergo nutritional changes by virtue of a plasmic circulation in their tubular structure, and two of their hard tissues manifest sensibility, which in disease may be exalted to extreme painfulness. The teeth are not parts of the true skeleton, but may be considered as elements of a dermal skeleton. They are developed from the tegument of the mouth, and though closely embraced by the alveolar processes of the maxillary bones, are never in man united to them." While we cannot in every case trace this ridged, pitted, or honeycombed condition of the teeth to the presence of serious illness of the patient during the time when the defective portions of the teeth were being developed, it can scarcely be doubted that an imperfect organization of the teeth, if not the result of some special disease, such as measles, influencing the system generally, is yet consequent upon constitutional conditions. The simple fact that if one tooth is affected those parts of other teeth which correspond in respect to the period of formation will present a similar condition, precludes the supposition that the effect is due merely to a local cause. Hereditary syphilis in its effects may often be traced as the cause of a peculiarly dwarfed condition of certain teeth. The incisors and cuspids are of small size, and peg-shaped; the crowns are notched, the notch being in the main a concavity from the one corner to the other, though there may be secondary notches in this general concavity. Teeth of the class which we note as syphilitic have a dusky, opaque appearance, and are relatively small. They are of a very soft character, and consequently wear down very rapidly. Moreover, it is claimed by the best authorities that constitutional syphilis is capable of modifying the nutrition of many parts of the body, manifesting its influence in attacks on the hair, the nails, and the skin generally. Now the homological relation which exists between the teeth and various dermal appendages may serve, if not to explain, at least to render less surprising, the fact that the developing teeth should be a chosen site for its manifestation.

So much for the arrest of development. Now what are the effects of zymotic diseases upon teeth which bear none of the marks of partial development? We answer, that all febrile conditions or diseases predispose teeth to decay, first, by diminishing vitality, second, by changing the secretions of the mouth so that they act injuriously upon the teeth. In dyspepsia we have not only the predisposing but also the exciting causes of decay, as it not only impairs vitality, but prepares in the stomach an acid that is continually being thrown upon the teeth, which acts upon them with great energy. I need scarcely say that residence for any great length of time in miasmatic regions, inducing unfavorable conditions, institutes a predisposing cause, and in brief, all epidemic, endemic, dietic, and enthetic diseases derange the functions of the body, vitiate the secretions, impair the vitality, and may be classed as predisposing causes of decay.

